# Citizen Science Provides an Efficient Method for Broad-Scale Tick-Borne Pathogen Surveillance of Ixodes pacificus and Ixodes scapularis across the United States

**DOI:** 10.1128/mSphere.00682-21

**Published:** 2021-09-29

**Authors:** W. Tanner Porter, Julie Wachara, Zachary A. Barrand, Nathan C. Nieto, Daniel J. Salkeld

**Affiliations:** a Translational Genomics Research Institute, Flagstaff, Arizona, USA; b Northern Arizona University, Flagstaff, Arizona, USA; c Colorado State University, Fort Collins, Colorado, USA; University of California, Davis

**Keywords:** *Borrelia burgdorferi*, *Borrelia miyamotoi*, *Anaplasma phagocytophilum*, *Babesia microti*, tick-borne, *Borrelia*, relapsing fever, Lyme disease, anaplasmosis, babesiosis

## Abstract

Tick-borne diseases have expanded over the last 2 decades as a result of shifts in tick and pathogen distributions. These shifts have significantly increased the need for accurate portrayal of real-time pathogen distributions and prevalence in hopes of stemming increases in human morbidity. Traditionally, pathogen distribution and prevalence have been monitored through case reports or scientific collections of ticks or reservoir hosts, both of which have challenges that impact the extent, availability, and accuracy of these data. Citizen science tick collections and testing campaigns supplement these data and provide timely estimates of pathogen prevalence and distributions to help characterize and understand tick-borne disease threats to communities. We utilized our national citizen science tick collection and testing program to describe the distribution and prevalence of four *Ixodes-*borne pathogens, Borrelia burgdorferi *sensu lato*, Borrelia miyamotoi, Anaplasma phagocytophilum, and Babesia microti, across the continental United States.

**IMPORTANCE** In the 21st century, zoonotic pathogens continue to emerge, while previously discovered pathogens continue to have changes within their distribution and prevalence. Monitoring these pathogens is resource intensive, requiring both field and laboratory support; thus, data sets are often limited within their spatial and temporal extents. Citizen science collections provide a method to harness the general public to collect samples, enabling real-time monitoring of pathogen distribution and prevalence.

## INTRODUCTION

Tick-borne diseases (TBDs) have seen dynamic changes and increased incidence across the last 2 decades (1). These changes have been spurred by the discovery of new pathogens and vector or pathogen expansion (1). In the United States, two tick species are responsible for the preponderance of tick-borne diseases: the black-legged tick (Ixodes scapularis) east of the Rocky Mountains and the western black-legged tick (Ixodes pacificus) west of the Rocky Mountains (2). Both of these tick species harbor and transmit Borrelia burgdorferi *sensu lato*, Borrelia miyamotoi, and Anaplasma phagocytophilum; additionally, I. scapularis also transmits Babesia microti and Powassan virus (2). These pathogens are maintained in wildlife host populations, and tick-borne disease cases in humans result from spillover from these wildlife populations via a tick bite (2).

The incidence of tick-borne diseases is a culmination of complex transmission processes: reservoir host distribution, tick distribution, spatial ecology of pathogen prevalence in ticks and vertebrates, and facets of human-tick exposure (seasonality of behavior, risk factors for exposure, susceptibility to infection, etc.). These factors are often described by surveillance of ticks or reservoir hosts in the field. Field collections can provide high-resolution spatial data for reservoir or tick distributions and pathogen prevalence (3–6). However, this approach can be logistically expensive, time-consuming, and reliant on the availability and motivation of personnel; consequently, data can be geographically or temporally limited. Importantly, field collections may not accurately characterize human-tick exposure, a key component to understanding and predicting TBDs, if surveillance does not reflect variation in patterns of human use. For example, human recreation patterns may not synchronize with tick phenology patterns, so exposure to ticks increases when tick abundance decreases (7), or surveillance may neglect particular habitats in favor of sites or habitats that can guarantee reliable samples (8).

Tick-borne pathogen surveillance in human populations can be achieved through disease reporting. In the United States, the Centers for Disease Control and Prevention’s (CDC’s) National Notifiable Diseases Surveillance System (NNDSS) records cases of several TBDs (9), and this system enables us to understand general disease trends (e.g., distribution, seasonality, and abundance). Similar to field surveillance of ticks and reservoir hosts, there are disadvantages to this approach, including the following. (i) Spatial data are reported as the patient’s county of residence rather than the location of exposure (10). (ii) Not all states participate equally (e.g., different states have different disease reporting guidelines). Many patients are probably exposed within their county of residence, either peridomestically or while recreating in nearby natural areas (7, 11); however, people also travel and may be exposed to the tick and its pathogen in areas that are not accurately captured by the county of residence. Furthermore, the system relies on conscientious reporting of diagnosed cases, but recent estimates suggest a much larger burden of tick-borne diseases than the NNDSS system reports (12–14).

Citizen science—when members of the public voluntarily collaborate with scientists to collect data and samples—offers a third approach to augment our knowledge of vector-borne disease epidemiology (15–19). Citizen science-based collection efforts can opportunistically collect and test ticks from broad spatial scales, reasonably cheaply and quickly, and hone in on elements of human-tick exposures. These benefits are leveraged by harnessing citizen scientists’ interest in collecting and submitting ticks they encounter, therefore providing a method to supplement traditional surveillance methods that might be conducted at restricted temporal or spatial extents. The accuracy and validity of citizen science data are limited by their nature (e.g., lack of sampling control and verification), and in some cases, these limitations mirror those found in human disease reporting or field collections: namely, the unknown certainty of exposure sites and a lack of spatial and temporal uniformity in surveillance effort (8, 10, 18, 20). Despite these possible drawbacks, this approach can undoubtedly augment traditional surveillance techniques and provide valuable insights into TBD ecology and epidemiology. Additionally, citizen science collections can efficiently monitor traditionally nonendemic areas for pathogen emergence as these areas are rarely monitored through traditional active surveillance campaigns (20).

This study utilized our national citizen science tick collection program to investigate the pathogen distribution and prevalence of Anaplasma phagocytophilum, Babesia microti, Borrelia burgdorferi *sensu lato* (Lyme group *Borrelia*), and Borrelia miyamotoi across the United States.

## RESULTS

### Tick collections.

**FIG 1:**
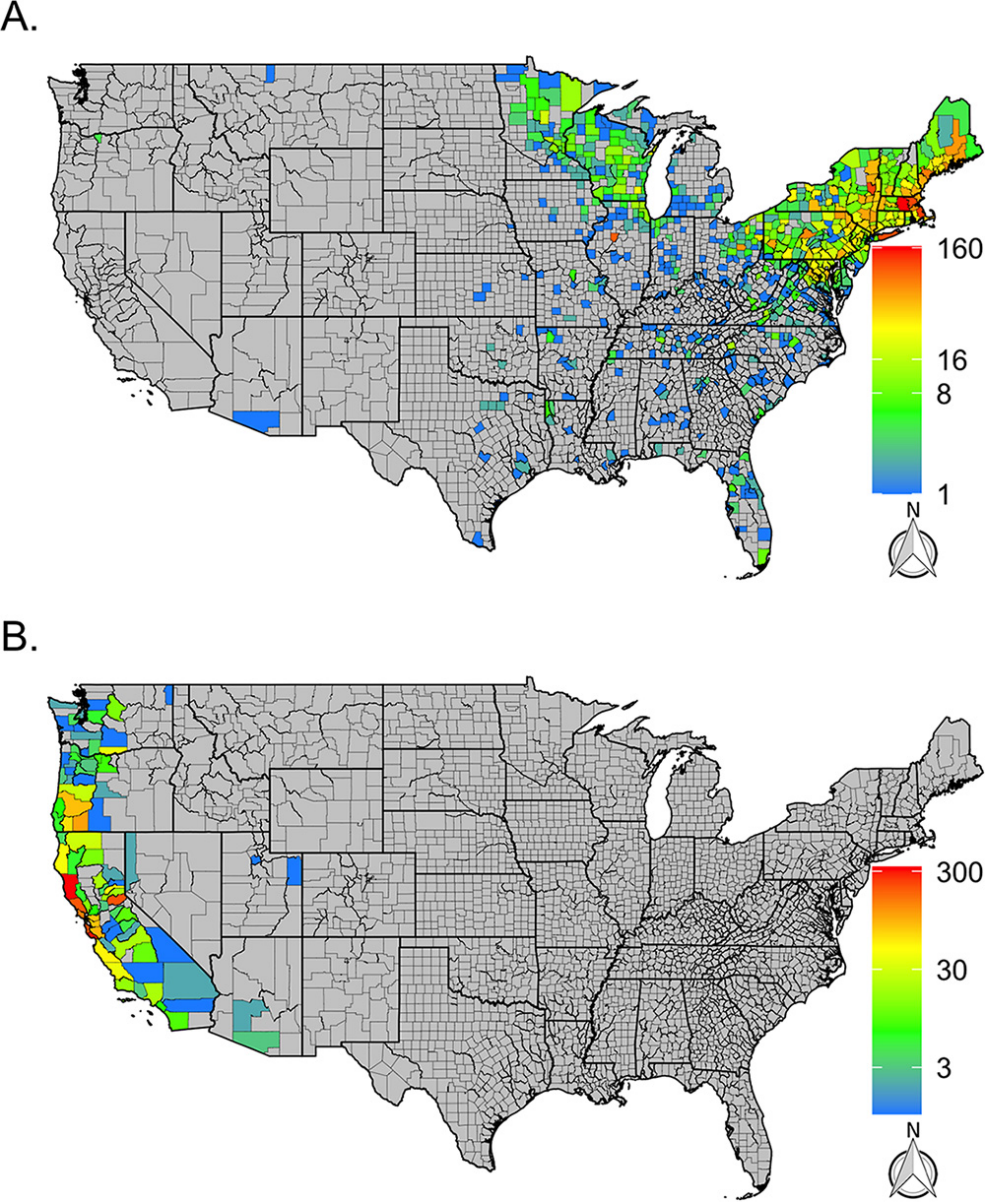
Distribution and number of I. scapularis (A) and I. pacificus (B) collected through the citizen science program from 2016 to 2019 by county. Gray counties indicate counties without tick submissions, and in both panels, color scales are individually calibrated.

A total of 6,429 I. scapularis ticks (larvae, 178; nymphs, 1,894; adults, 4,334; unknown life stage, 23) and 2,525 I. pacificus ticks (larvae, 18; nymphs, 271; adults, 2,227; unknown life stage, 9) were collected from across the known species ranges (18, 21). I. scapularis ticks were received from 692 different counties with a mean of 9.3 ticks per county (range, 1 to 164; median, 2.5). Similarly, I. pacificus ticks were received from 87 counties with a mean of 29.4 ticks per county (range, 1 to 332; median, 4.5). As expected, submissions of I. scapularis were concentrated to counties in the Northeast and upper Midwest, with sporadic submissions from the southern counties (Fig. 1A). Additionally, a few submissions were received from counties outside these areas, including Arizona, Oregon, and Montana. Submissions of I. pacificus were concentrated to counties on the west coast (California, Oregon, and Washington) (Fig. 1B).

**FIG 2:**
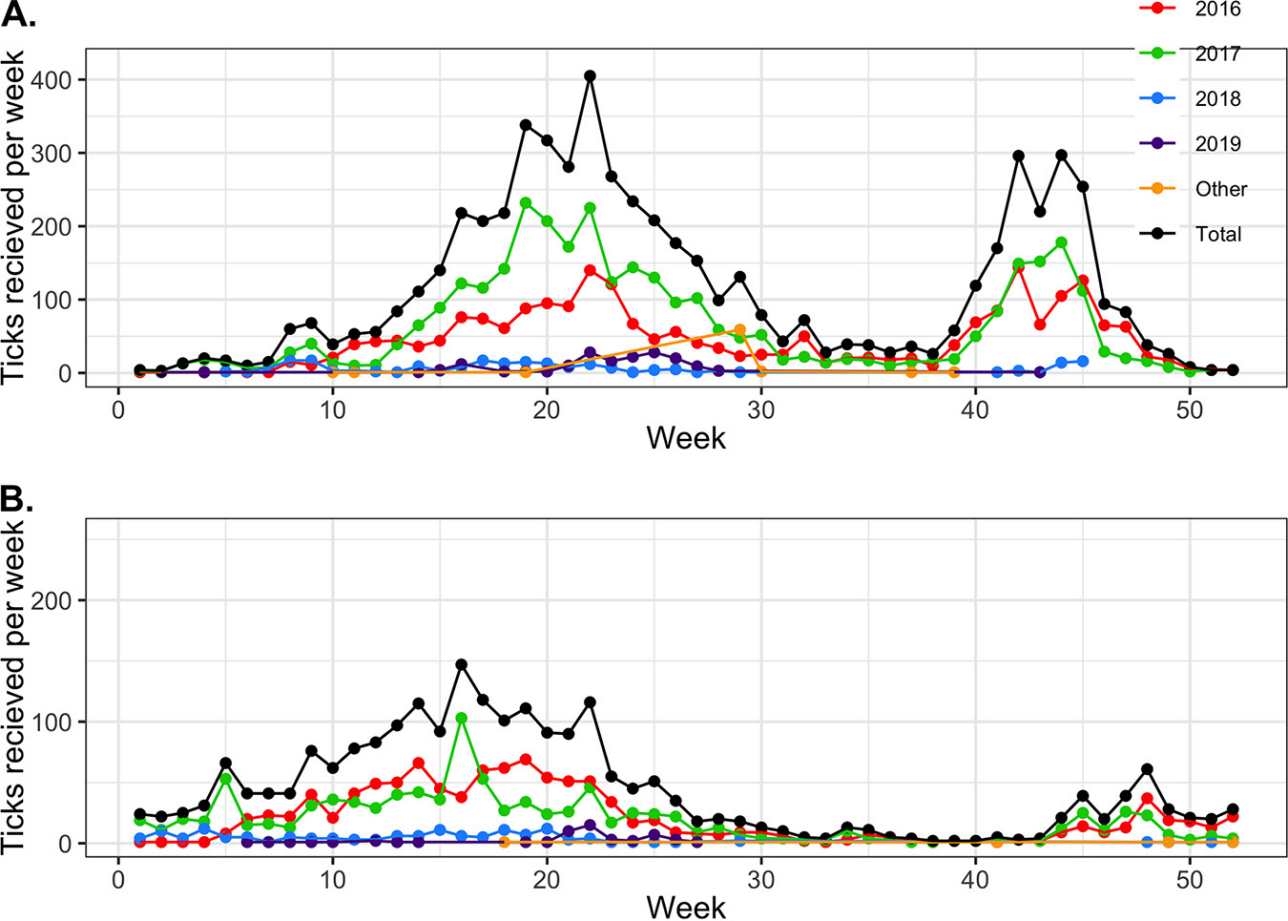
Number of I. scapularis (A) and I. pacificus (B) ticks collected by CDC week (Sunday to Saturday) and across years.

The highest rate of submissions in 2016 and 2017 was encountered from early April to late June and late September to early November of each year (Fig. 2). Further breakdowns of temporal patterns of submissions can be found in our previous studies (7, 11, 18).

Besides I. scapularis and I. pacificus, a total of six Ixodes cookei nymphs, one adult Ixodes kingi, one adult Ixodes neotomae, one Ixodes ricinus nymph (travel associated), and one adult Ixodes spinipalpis were received. I. cookei ticks were received from two counties (Albany, NY, and Gilmer, WV), each with three submissions. Except for I. ricinus, all of these ticks tested negative for all pathogens. The *I. ricinus* submission form noted previous travel history to Europe, and the single tick tested positive for Lyme group *Borrelia.*

### Pathogen distribution.

**FIG 3:**
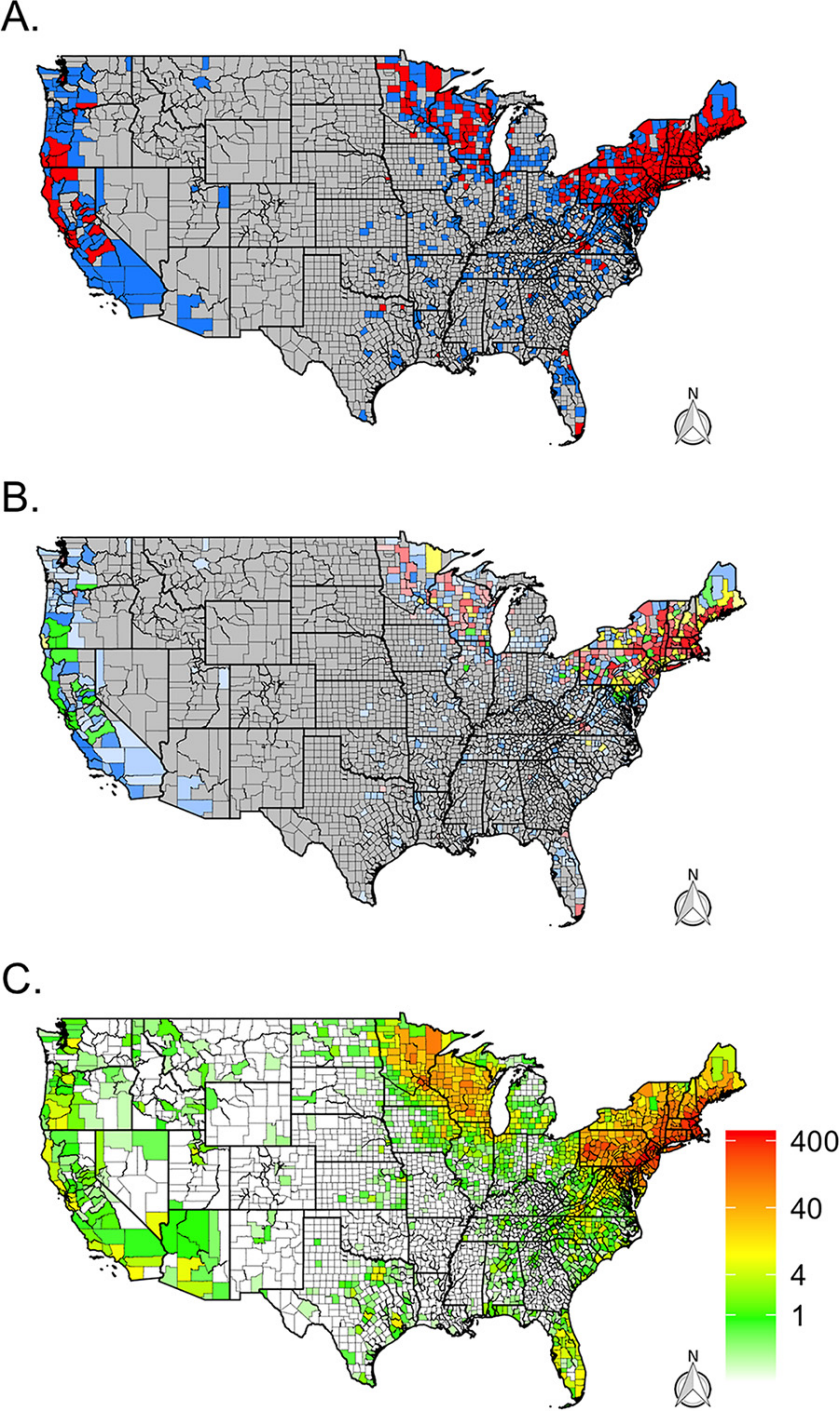
Distribution of Lyme group *Borrelia* (B. burgdorferi sensu lato) and Lyme disease cases across the continental United States. (A) Presence (red) and absence (blue) via real-time PCR for B. burgdorferi sensu lato by county. Gray counties indicate counties without tick submissions. (B) Prevalence of positive *Ixodes* ticks via real-time PCR for B. burgdorferi sensu lato by county. Gray counties indicate no tick submissions, blue counties indicate no positive ticks, green indicates a tick prevalence between 0 and 10%, yellow indicates a prevalence between 10 and 20%, and red indicates a prevalence greater than 20%. The color’s opacity indicates the estimate’s confidence, with darker opacity indicating a smaller confidence interval, while lighter opacities indicate wider confidence intervals. (C) Average number of human Lyme disease cases per year and county reported to CDC’s National Notifiable Disease Surveillance System between 2009 and 2018.

Overall, Lyme group *Borrelia* was identified in 1,279 (14%) ticks submitted from 293 counties across the range of I. scapularis and I. pacificus. These counties were concentrated in the Northeast, upper Midwest, and northern California (Fig. 3A). Lyme group *Borrelia* was found in 75% of all counties and in 94% of counties with more than five submitted I. scapularis ticks in the Northeastern United States and 78% of counties with more than five submitted I. scapularis ticks in the Midwest (Table 1). In the West, Lyme group *Borrelia* was detected in 26% of the surveyed counties (number of *Ixodes *> 0) and 15% of the surveyed counties in the southern United States. However, the prevalence of Lyme group *Borrelia* varied by region. In the Northeast, Lyme group *Borrelia* was detected in 23% of total submitted I. scapularis ticks (Table 2). It was common to see high prevalence in counties across the region (Fig. 3B). In the West, Lyme group *Borrelia* was found in 3% of submitted I. pacificus ticks.

**TABLE 1:** Percentage of counties by region with detected A. phagocytophilum, Babesia microti, Lyme group *Borrelia*, and B. miyamotoi ticks across the United States using citizen science collections

Pathogen	Region	Tick species	Regional distribution				
			% counties with *Ixodes *> 0 (no. of counties/ total no. of counties)	% counties with *Ixodes > *5 (no. of counties/ total no. of counties)	% counties with *Ixodes *> 10 (no. of counties/ total no. of counties)	% counties with *Ixodes *> 20 (no. of counties/ total no. of counties)	% counties including counties without submissions (no. of counties/ total no. of counties)
A. phagocytophilum	West	I. pacificus	14 (12/86)	24.4 (10/41)	31 (9/29)	45 (9/20)	2.7 (12/448)
	Midwest	I. scapularis	7.4 (18/243)	19.5 (8/41)	27.8 (5/18)	20 (1/5)	1.7 (18/1,054)
	Northeast	I. scapularis	43.7 (90/206)	58.4 (87/149)	70.3 (78/111)	83.6 (61/73)	41.5 (90/217)
	South	I. scapularis	3.3 (8/240)	14.3 (5/35)	28.6 (4/14)	33.3 (2/6)	0.6 (8/1,417)

*Bab. microti*	West	I. pacificus	0 (0/86)	0 (0/41)	0 (0/29)	0 (0/20)	0 (0/448)
	Midwest	I. scapularis	5.8 (14/243)	17.1 (7/41)	11.1 (2/18)	0 (0/5)	1.3 (14/1,054)
	Northeast	I. scapularis	25.2 (52/206)	34.2 (51/149)	43.2 (48/111)	58.9 (43/73)	24 (52/217)
	South	I. scapularis	2.1 (5/240)	5.7 (2/35)	14.3 (2/14)	16.7 (1/6)	0.4 (5/1,417)

Lyme group *Borrelia*	West	I. pacificus	25.6 (22/86)	51.2 (21/41)	62.1 (18/29)	80 (16/20)	4.9 (22/448)
	Midwest	I. scapularis	30.5 (74/243)	78 (32/41)	83.3 (15/18)	100 (5/5)	7 (74/1,054)
	Northeast	I. scapularis	78.6 (162/206)	94 (140/149)	97.3 (108/111)	100 (73/73)	74.7 (162/217)
	South	I. scapularis	14.6 (35/240)	51.4 (18/35)	71.4 (10/14)	100 (6/6)	2.5 (35/1,417)

B. miyamotoi	West	I. pacificus	20.9 (18/86)	41.5 (17/41)	48.3 (14/29)	70 (14/20)	4 (18/448)
	Midwest	I. scapularis	4.1 (10/243)	12.2 (5/41)	16.7 (3/18)	40 (2/5)	0.9 (10/1,054)
	Northeast	I. scapularis	22.3 (46/206)	27.5 (41/149)	34.2 (38/111)	46.6 (34/73)	21.2 (46/217)
	South	I. scapularis	2.5 (6/240)	8.6 (3/35)	21.4 (3/14)	33.3 (2/6)	0.4 (6/1,417)

**TABLE 2:** Average county prevalence by region of A. phagocytophilum, *Bab. microti*, Lyme group *Borrelia*, and B. miyamotoi across the United States using citizen science collections

Pathogen	Region	Tick species	Avg county prevalence				Prevalence by region, % colonies (no. of counties/ total no. of counties)
			% all counties (no. of counties) [SD, range]	% counties with *Ixodes > *5 (no. of counties) [SD, range]	% counties with *Ixodes* > 10 (no. of counties) [SD, range]	% counties with *Ixodes* > 20 (no. of counties) [SD, range]	
A. phagocytophilum	West	I. pacificus	1.6 (67) [7, 0−50]	0.8 (41) [2.7, 0−16.7]	0.6 (29) [1, 0−4.3]	0.8 (20) [1.2, 0−4.3]	1.2 (30/2,525)
	Midwest	I. scapularis	3.1 (144) [9.8, 0−50]	3.2 (41) [7.9, 0−33.3]	2.8 (18) [4.9, 0−15]	1.6 (5) [3.5, 0−7.9]	3.2 (33/1,035)
	Northeast	I. scapularis	4.1 (192) [6.2, 0−33.3]	4.7 (149) [5.7, 0−24.4]	5.1 (111) [5.1, 0−24.4]	5.2 (73) [4.5, 0−24.4]	5.3 (238/4,533)
	South	I. scapularis	1.1 (117) [5.2, 0−33.3]	1.1 (35) [3.3, 0−16.7]	1.6 (14) [2.8, 0−7.7]	1.2 (6) [2, 0−4.8]	0.9 (8/852)

*Bab. microti*	Midwest	I. scapularis	1.6 (144) [6.6, 0−50]	2.2 (41) [5.3, 0−22.2]	0.9 (18) [2.6, 0−9.1]	0 (5) [0, 0−0]	1.4 (15/1,035)
	Northeast	I. scapularis	1.5 (192) [3.6, 0−25]	1.8 (149) [3.5, 0−25]	2 (111) [3.1, 0−16.7]	2.4 (73) [2.8, 0−14.3]	2.1 (97/4,533)
	South	I. scapularis	0.5 (117) [3.6, 0−33.3]	0.2 (35) [1.2, 0−6.7]	0.6 (14) [1.8, 0−6.7]	0.3 (6) [0.8, 0−1.9]	0.6 (5/852)

Lyme group *Borrelia*	West	I. pacificus	2.6 (67) [7.2, 0−50]	3 (41) [5.1, 0−25]	2.5 (29) [2.8, 0−10]	2.9 (20) [2.3, 0−7.1]	2.9 (73/2,525)
	Midwest	I. scapularis	16 (144) [23.2, 0−100]	16.9 (41) [14.3, 0−50]	13.3 (18) [10.6, 0−31.6]	7.3 (5) [6, 1.5−14.8]	12.7 (131/1,035)
	Northeast	I. scapularis	19.9 (192) [15.3, 0−100]	21.7 (149) [12.5, 0−66.7]	22.3 (111) [11.4, 0−64]	24 (73) [10, 4−64]	22.6 (1,024/4,533)
	South	I. scapularis	5.8 (117) [14.4, 0−100]	6.5 (35) [8.1, 0−25]	6.8 (14) [7.3, 0−21.4]	7.7 (6) [6.4, 1.9−19]	6 (51/852)

B. miyamotoi	West	I. pacificus	1.8 (67) [6.7, 0−50]	1.8 (41) [3.6, 0−16.7]	1.1 (29) [1.7, 0−6]	1.7 (20) [1.8, 0−6]	1.5 (37/2,525)
	Midwest	I. scapularis	1 (144) [5.4, 0−50]	0.9 (41) [2.6, 0−11.1]	0.7 (18) [1.8, 0−6.2]	1.3 (5) [1.9, 0−3.7]	1.1 (11/1,035)
	Northeast	I. scapularis	1.5 (192) [4.3, 0−25]	1.1 (149) [2.3, 0−12.5]	1.2 (111) [2, 0−9.1]	1.4 (73) [1.9, 0−7.1]	1.3 (59/4,533)
	South	I. scapularis	0.3 (117) [2.4, 0−25]	0.3 (35) [1.2, 0−5]	0.9 (14) [1.8, 0−5]	1.2 (6) [2, 0−4.8]	0.7 (6/852)

**FIG 4:**
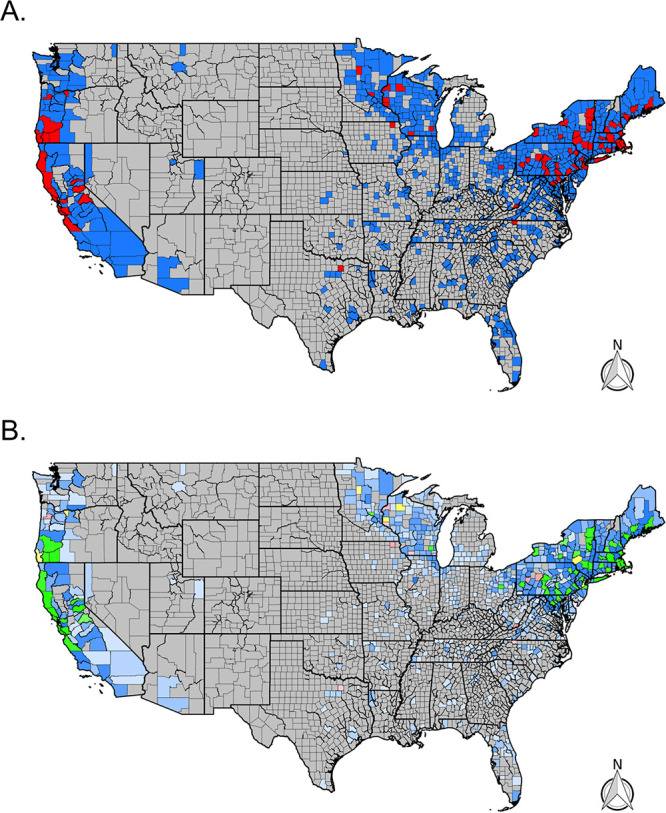
Distribution of B. miyamotoi (TBRF *Borrelia)* across the continental United States. (A) Presence (red) and absence (blue) via real-time PCR for B. miyamotoi. Gray counties indicate counties without tick submissions. (B) Prevalence of positive *Ixodes* ticks via real-time PCR for B. miyamotoi by county. Gray counties indicate no tick submissions, blue counties indicate no positive ticks, green indicates a tick prevalence between 0 and 10%, yellow indicates a prevalence between 10 and 20%, and red indicates a prevalence greater than 20%. The color’s opacity indicates the estimate's confidence, with darker opacity indicating a smaller confidence interval, while lighter opacities indicate wider confidence intervals.

B. miyamotoi was identified in 113 ticks (1%) submitted from 80 total counties. The majority of these counties were in the northeastern United States or California (Fig. 4A), where 28% (Northeast) and 42% (West) of counties with more than five submitted *Ixodes* ticks tested positive for B. miyamotoi. In addition, sporadic detection of B. miyamotoi was detected in southern counties where 2.5% of surveyed counties had at least one infected tick. In the Midwest, 4% of surveyed counties had at least one infected tick. Overall, the prevalence of B. miyamotoi varied and was 1.5% in the West, 1.1% in the Midwest, 1.3% in the Northeast, and 0.7% in the South (Fig. 4B and Table 2).

**FIG 5:**
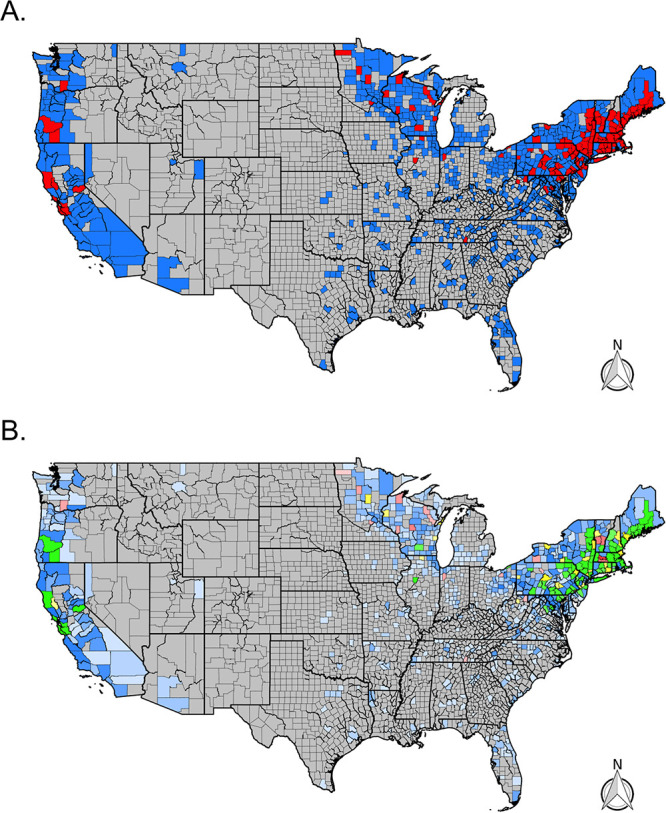
Distribution of A. phagocytophilum across the continental United States. (A) Presence (red) and absence (blue) via real-time PCR for A. phagocytophilum by county. Gray counties indicate counties without tick submissions. (B) Prevalence of positive *Ixodes* ticks via real-time PCR for A. phagocytophilum by county. Gray counties indicate no tick submissions, blue counties indicate no positive ticks, green indicates a tick prevalence between 0 and 10%, yellow indicates a prevalence between 10 and 20%, and red indicates a prevalence greater than 20%. The color’s opacity indicates the estimate's confidence, with darker opacity indicating a smaller confidence interval, while lighter opacities indicate wider confidence intervals.

Across the Northeast, A. phagocytophilum was detected in 42% of counties and in 58% of counties with more than five submitted I. scapularis ticks (Table 1). A. phagocytophilum was detected in 20% of Midwestern counties and 24% of Western counties with more than five *Ixodes* submissions (Fig. 5A and Table 1). In total, 309 (3%) *Ixodes* ticks from 128 counties had detectable A. phagocytophilum. The regional prevalence of A. phagocytophilum was highest in the Northeast (5.3%), followed by the Midwest (3.2%) and West (1.2%) (Fig. 5B and Table 2).

**FIG 6:**
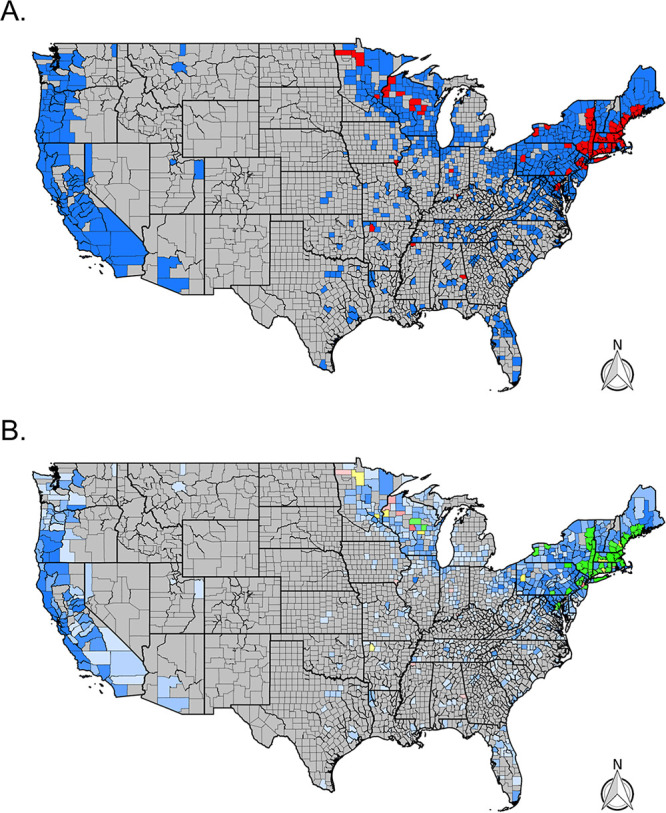
Distribution of Babesia microti across the continental United States. (A) Presence (red) and absence (blue) via real-time PCR for *Bab. microti* by county. Gray counties indicate counties without tick submissions. (B) Prevalence of positive *Ixodes* ticks via real-time PCR for *Bab. microti* by county. Gray counties indicate no tick submissions, blue counties indicate no positive ticks, green indicates a tick prevalence between 0 and 10%, yellow indicates a prevalence between 10 and 20%, and red indicates a prevalence greater than 20%. The color’s opacity indicates the estimate’s confidence, with darker opacity indicating a smaller confidence interval, while lighter opacities indicate wider confidence intervals.

Babesia microti was identified in 117 (2%) Ixodes scapularis ticks from 71 counties. In the Northeast, 34% of counties with more than five submitted I. scapularis had at least one infected tick. Similarly, in the Midwest and South, 20% (Midwest) and 6% (South) of counties with more than five submitted I. scapularis had infected ticks (Fig. 6A). Regional *Bab. microti* prevalence ranged from 2.1% (Northeast) to 0.6% (South) (Fig. 6B and Table 2). *Bab. microti* was not detected in any I. pacificus ticks.

In general, average county prevalence across each region was similar to the total prevalence estimate; however, adding criteria for inclusion based on the number of submitted ticks (e.g., number of *Ixodes *> 5, 10, or 20) into the average prevalence calculation created estimates with lower standard deviations and ranges as the required sample size increased and counties with minimal submissions were removed (Table 2). Individual county data are available as a supplemental table (see Table S1 in the supplemental material).

10.1128/mSphere.00682-21.1 TABLE S1 County level pathogen data for I. scapularis and I. pacificus. Download Table S1, CSV file, 0.09 MB. Copyright © 2021 Porter et al. 2021 Porter et al. https://creativecommons.org/licenses/by/4.0/ This content is distributed under the terms of the Creative Commons Attribution 4.0 International license.

### Pathogen sequencing.

A total of 184 (14%) Lyme group *Borrelia*-positive samples were sequenced from across the country (Northeast, 150; Midwest, 12; South, 5; West, 11) (Table 3). In the Northeast, sequenced samples were collected from Connecticut, Massachusetts, Maine, New Hampshire, New Jersey, New York, Pennsylvania, Rhode Island, and Vermont, with 100% (150/150) of the sequences aligned with Borrelia burgdorferi *sensu stricto*. In the Midwest, sequenced samples were collected from Illinois, Michigan, Minnesota, Ohio, and Wisconsin; similarly, in the Northeast, 100% (12/12) of the sequences aligned with B. burgdorferi *sensu stricto*. In the South, sequenced samples were collected from Georgia (*n* = 1), Maryland (*n* = 2), and Virginia (*n* = 2). All sequences from Maryland and Virginia (4/4) aligned with B. burgdorferi *sensu stricto*. The sample from Georgia aligned with Borrelia andersonii. In the West, samples were sequenced from California (*n* = 10) and Washington (*n* = 1), of which the majority (10/11) of sequences aligned with B. burgdorferi *sensu stricto*. Additionally, one Lyme group *Borrelia*-positive sample from California aligned with Borrelia bissettiae.

**TABLE 3:** Results of sequence analysis by pathogen and U.S. region

Pathogen	No. of samples sequenced				Total no. of samples sequenced
	Midwest	Northeast	South	West	
A. phagocytophilum	6	20	0	0	26
*B. andersonii*	0	0	1	0	1
*B. bissettiae*	0	0	0	1	1
B. burgdorferi *sensu stricto*	12	150	4	10	176
B. miyamotoi	2	12	0	16	30

Similarly, a total of 30 (27%) positive B. miyamotoi samples were sequenced from the Midwest (*n* = 2), Northeast (*n* = 12), and West (*n* = 16) (Table 3). Again, all samples aligned with B. miyamotoi. Finally, 26 (8%) A. phagocytophilum samples were sequenced from the Northeast (*n* = 20) and Midwest (*n* = 6), of which all sequences aligned with A. phagocytophilum (Table 3).

## DISCUSSION

### Vector distribution.

**FIG 7:**
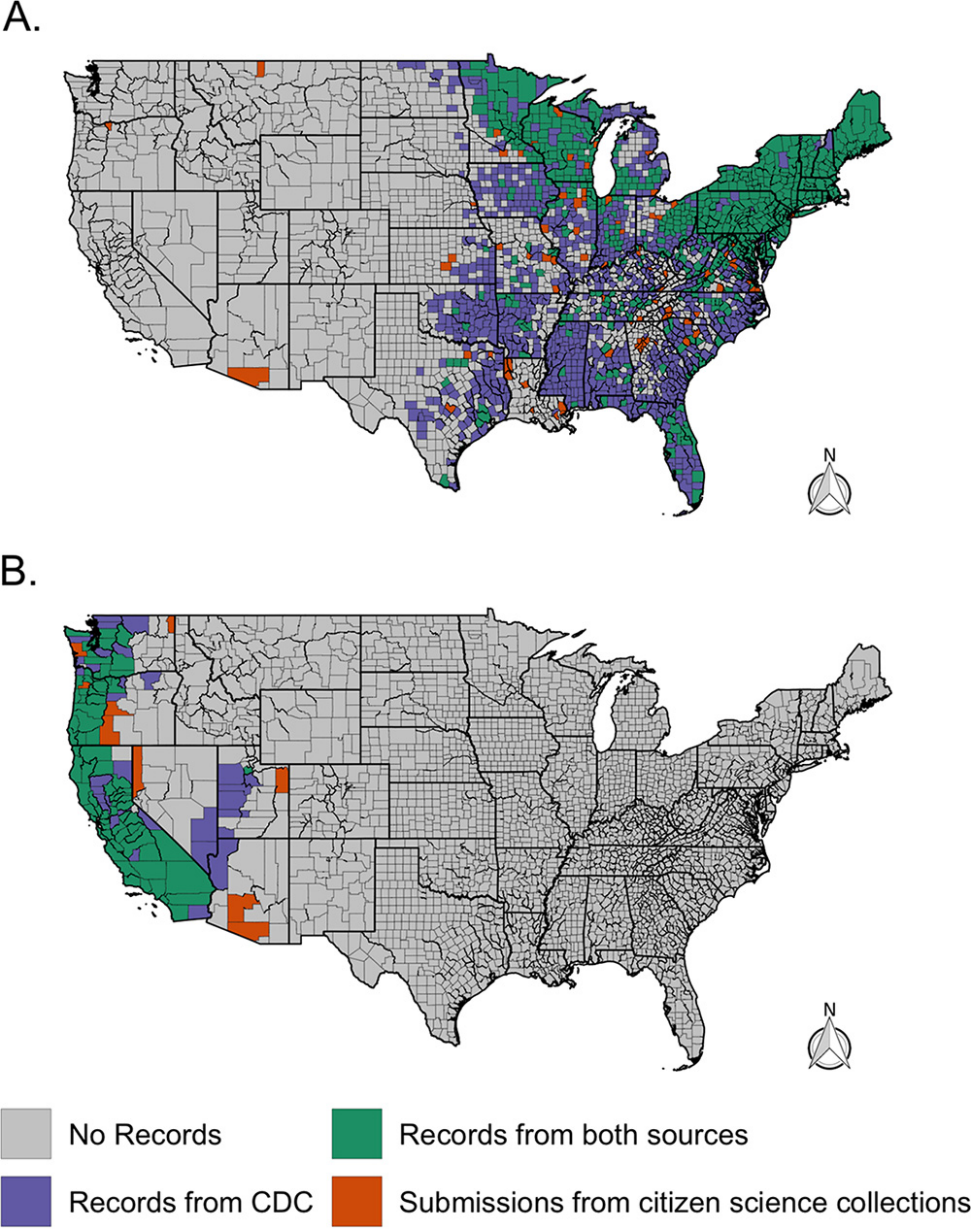
County-level comparison of I. scapularis (A) and I. pacificus (B) distributions from a recent CDC report (23) and citizen science tick collections. Counties that had reported (established or reported) I. scapularis or I. pacificus populations were recorded as present (blue or green).

Our citizen science-based collection of *Ixodes* ticks was able to characterize tick and pathogen distribution patterns across large portions of the United States, with tick submissions received over just 4 years. Our trends are similar to reported cases of Lyme disease (Fig. 3C) (22) and the CDC map (23); citizen science submissions showed *Ixodes* foci in the Northeast, upper Midwest, and far-western United States (Fig. 7). In the southeastern United States, citizen science data revealed fewer counties than the CDC map with Ixodes scapularis populations (Fig. 7A); this may reflect fewer human-tick exposures in this area versus concerted field surveillance efforts to locate ticks. In addition, most counties with citizen science I. scapularis observations that differed from the CDC map were located near counties currently recognized to have tick populations. However, some counties were located further away (Montana, Oregon, and Arizona) and can best be explained as travel-associated tick exposures. Similar lessons are apparent for the western black-legged tick, I. pacificus: discrepancies between CDC maps and citizen surveillance were infrequent, and most counties that we received ticks from are nearby counties that have established populations (Fig. 7B).

For both tick species, the “new” county records should not be regarded as having “confirmed” established tick populations, in part because the travel history of the submitting citizen scientists was not verified. However, these data should also not be regarded as surprising: they often mirror established knowledge and may simply be filling in existing data gaps; they can be used as information on where ticks are biting people, and they can generate interest and impetus to conduct field surveillance in those locales.

### *Borrelia* pathogen distributions.

**FIG 8:**
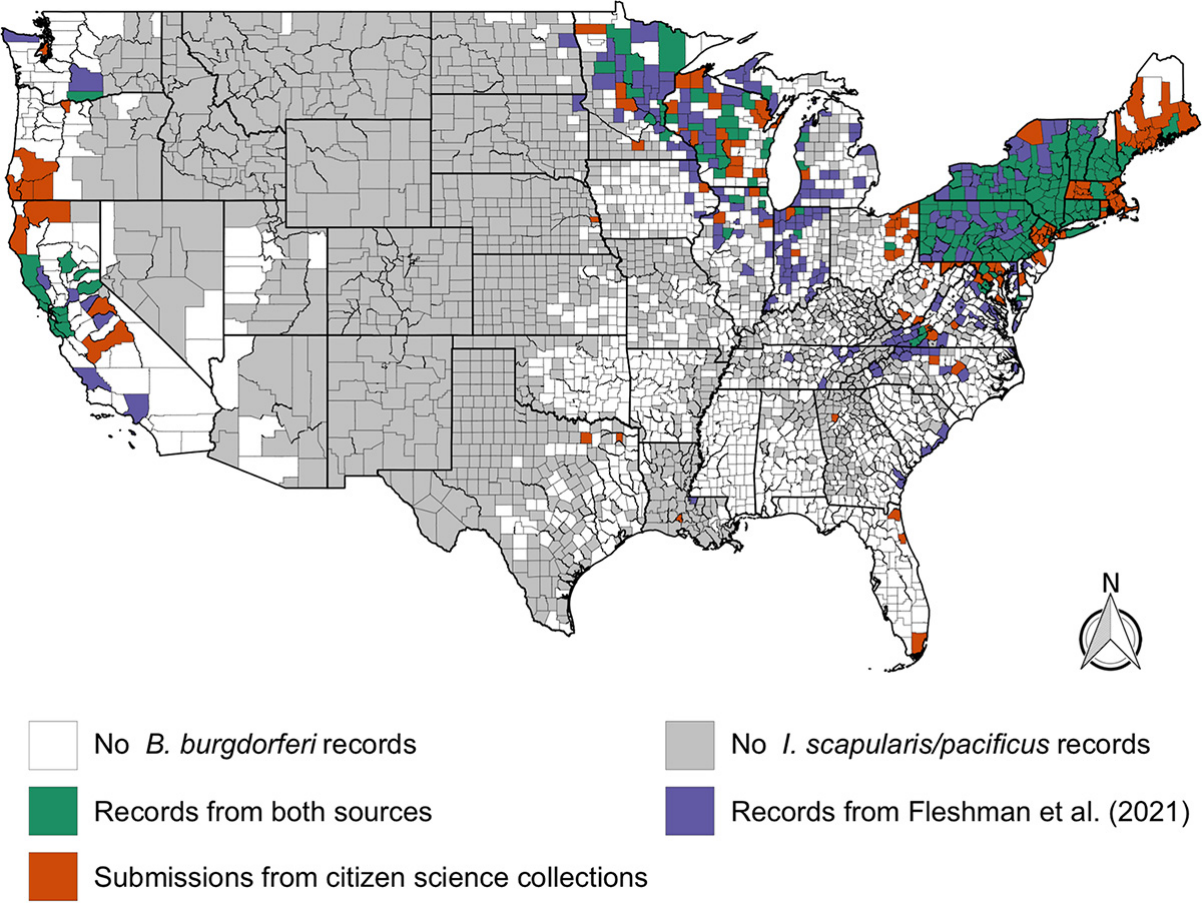
County-level comparison of B. burgdorferi distributions from Fleshman et al. (5) (B. burgdorferi *sensu stricto*) and citizen science pathogen testing results (B. burgdorferi sensu lato).

The *Borrelia* pathogen data elicited by the citizen science project generally followed the distribution of I. scapularis and I. pacificus across the United States. Comparing B. burgdorferi sensu lato trends to previous work finds similar trends (5). However, several caveats need to be described before comparing our B. burgdorferi sensu lato maps to Fleshman et al. (5) (Fig. 8). First, Fleshman et al. (5) restricted a county’s presence/absence status only to B. burgdorferi *sensu stricto* in ticks collected by scientists. Our data are for Lyme group *Borrelia*—and so may incorporate more diversity in tick-borne pathogens.

Given these differences, it is remarkable how the citizen science-generated data provide a very similar overview of the geographic distribution of B. burgdorferi (Fig. 8). Both approaches identified the main Lyme disease strongholds of the Northeast and the upper Midwest. However, in places like southwestern Virginia, the data from field surveillance and citizen science surveillance are strikingly complementary. Similarly, there are sporadic, seemingly isolated counties in Florida, Georgia, South Carolina, and even Louisiana where B. burgdorferi sensu lato infections are observed. Looking at both data sets, it would seem that local ecology and epidemiology of B. burgdorferi sensu lato ought to merit further study in states outside the well-known endemic foci.

On the West Coast, though overall patterns of *Borrelia* infection in I. pacificus are largely consistent between studies, there are interesting discrepancies. For example, Humboldt County, California, is designated as not having B. burgdorferi based on field surveillance (5). However, citizen scientist submissions suggest that B. burgdorferi sensu lato is locally present. Though this could be an anomaly, and the citizens resident in Humboldt County may have encountered B. burgdorferi sensu lato-positive ticks during travels, B. burgdorferi is known to circulate in mammal communities in Humboldt County (24). In this case, relying on the publication of field-collected I. pacificus data since 2000 misleadingly portrays B. burgdorferi as absent from the county. A similar issue may account for the discrepancy between maps for southern Oregon, where results on local B. burgdorferi sensu lato have not been published or may not have been conducted. These cases illustrate the scenario where citizen science data could inform field surveillance efforts to confirm or reject local tick-borne disease endemism hypotheses.

The presence and absence of tick-borne pathogens is one way of presenting surveillance results, but it is binary and can confound interpretation of the likelihood of exposure to a disease, e.g., if the pathogen is rare or frequent, it will be reported identically in a presence/absence map. As an illustration, Los Angeles County can be described as having B. burgdorferi *sensu stricto* present (5), but B. burgdorferi is extremely rare in southern California (e.g., 1/5,571 = 0.02%) (8, 25–27).

Prevalence—the proportion of infected ticks—may also help determine local tick-borne disease ecology and epidemiology with additional nuanced patterns (Fig. 1B, 2B, 3B, and 4B). However, prevalence can also be misleading as sample sizes can skew these estimates and the scale of data aggregation (e.g., county, state, region) (8), though incorporating confidence intervals can help with interpretation. Small sample sizes that fail to find a pathogen (absence, zero prevalence) will generate large confidence intervals, as will small sample sizes with a few positive results (presence, but a conflated high prevalence). Larger sample sizes provide a more accurate illustration of pathogen presence/absence and prevalence. An argument could be made to impose some form of a threshold for the sample size to be reported, but the disadvantage is that data and information are then lost. One way to incorporate the uncertainties of existing data is to portray the confidence intervals within the maps, and we attempt this by using maps with differing opacity or transparency. In essence, this allows for counties with lower confidence in the prevalence estimates (smaller sample sizes) to be displayed by a lighter shade in the coloration. Similarly, counties with higher confidence in the prevalence estimates (larger sample size) are displayed using darker shades in the coloration.

### *Anaplasma* and *Babesia*.

A. phagocytophilum and Babesia microti distribution and prevalence generally reflected the NNDSS clinical case records; however, in some cases, the states that do not report these diseases to the NNDSS are within areas with significant pathogen distributions and prevalence (i.e., human babesiosis cases are not reported in Pennsylvania) (28). Overall, B. miyamotoi, A. phagocytophilum, and *Bab. microti* were detected at a lower prevalence compared to Lyme group *Borrelia*, with visual distributions often appearing spotty across states and regions. We hypothesize that the spotty pathogen distribution results from variable sample sizes due to the nature of nonstructured citizen science collections. A smaller sample size decreases the likelihood of pathogen detection, especially if the pathogen is rare. In addition, the number of ticks submitted is likely influenced by several components, e.g., heterogeneous tick densities, human-tick interactions, and knowledge/willingness to participate in citizen-based surveillance campaigns.

### Genotyping.

Lyme group *Borrelia* genotyping through DNA sequencing suggests that the presented Lyme group *Borrelia* (B. burgdorferi sensu lato) data have broadly estimated the prevalence of B. burgdorferi *sensu stricto*, the causative agent of Lyme disease, in the Northeast, upper Midwest, and West. These results are consistent with previous projects focusing on the genotyping of B. burgdorferi sensu lato, which has identified B. burgdorferi *sensu stricto* accounts for most isolates in northern California, the Midwest, and Northeastern United States (4, 8, 27, 29). However, these results must still be interpreted with caution, as a limited number of samples were sequenced in some regions. In addition to B. burgdorferi *sensu stricto* in California, we had a sample align with B. bissettiea—which has been previously observed in California (8, 29). Four of five samples that were sequenced from the South were from Maryland and Virginia and aligned with B. burgdorferi *sensu stricto*, which is similar to previous work (5). The additional sequenced sample was collected from Georgia and aligned with B. andersonii, a species previously detected in a patient in Georgia (30). The high diversity in Lyme group *Borrelia* in the South is consistent with previous findings (31, 32). All sequenced B. miyamotoi-positive samples aligned with B. miyamotoi samples, suggesting that the majority of the B. miyamotoi-positive samples are B. miyamotoi compared to other tick-borne relapsing fever (TBRF) spirochetes that have been found in hard ticks in the United States (e.g., Borrelia lonestari).

### Travel and uncertainty.

There are challenges associated with using citizen science collections; the challenges include submissions that are spatially misreported and uneven sampling, both of which add a level of uncertainty to these data (7, 11, 18). However, some of these challenges can be overcome through the incorporation of other data sets. Spatial uncertainty was evident in our program: several *Ixodes* submissions were submitted from outside their probable species range. A few I. scapularis submissions were received from the West Coast (Fig. 1A and 7A) and were likely the result of recent travel from areas where I. scapularis is endemic, a phenomenon that has been previously seen in citizen science collections (18, 19). We also received an *I. ricinus* submission—a tick endemic to Europe, but data associated with the tick submission confirmed that the tick was encountered during travel to France. Although no metadata was verified throughout the program after a tick was submitted, and travel history was not consistently collected, the I. scapularis and *I. ricinus* submissions serve as examples of easily detectable large-scale spatial inaccuracies (i.e., continent or national level). Citizen science collections can also create finer spatial uncertainties as well (i.e., state-state and county-county aberrations) that are harder to detect and quantify within the data set. Such spatial discordance can be easily attributed to county-to-county travel; however, they could also be evidence of tick/pathogen range expansions, especially when several submissions across time are received from a single county (20). In the future, we would advocate for a streamlined method to easily and accurately collect travel data associated with each submission to help limit these challenges. Even with these challenges, citizen science-based collections can characterize vector and pathogen distributions across large portions of the United States that compare favorably with active surveillance efforts (18, 20, 21) and broadly reflect clinical cases (22).

### Conclusion.

Citizen science provides a tool to complement pathogen monitoring in areas where tick-borne pathogens are endemic and areas where tick-borne pathogens are not endemic. These data must be carefully considered and interpreted because travel history may confound the geographical source of the infection or pathogen; similar issues arise with human case reports. Nonetheless, the benefits, especially when considered with the scale (nationwide), lack of expense (a fraction of active surveillance costs), and speed of data collection that a citizen science project can generate are extremely promising. Citizen science tick collections alone can provide interesting insights into a variety of qualitative tick and pathogen factors. However, citizen science data could be even more powerful if paired with traditional surveillance techniques (e.g., active surveillance) to synergistically increase sampling efficiency and address the dynamics of changing tick and tick-borne disease distributions. Additionally, citizen science pathogen monitoring can be expanded to other systems that could benefit from widespread and resource-efficient surveillance.

## MATERIALS AND METHODS

### Citizen science tick collection.

Ticks were collected through a free national tick identification and pathogen testing program at Northern Arizona University from 2016 to 2019 (7, 11, 18, 20). Detailed methods and descriptions are available (18); however, we briefly summarize the methods here. The accessibility of the program varied across the 4 years. The widest advertising and accessibility were available during 2016 and 2017 when the program operated without interruption. The program was initially advertised through a public relations campaign and was made available through a public website (Bay Area Lyme Foundation; https://www.bayarealyme.org/lyme-disease-prevention/tick-testing/), which became a top Internet result when the term “tick testing” was searched. Through this advertising, individuals and TBD awareness groups further advertised and shared the program. In 2018, the program was not advertised nor officially open; nonetheless, ticks were still submitted and tested. In 2019, the program operated from mid-June until November 1. Thus, submissions varied across years, influenced by advertising, awareness, and accessibility of the program. Ticks were submitted with a form that detailed the exposure location; no personal information was collected. The majority of submissions did not include recent travel information, and the research team did not verify the citizen scientist’s responses.

### Tick identification and molecular testing.

Ticks were identified to species, stage, and sex using morphological characteristics (33–35). If a sample was identified to the species level but the life stage was not identifiable, it was recorded as “unknown life stage.” Here we report data on I. scapularis and I. pacificus. Extracted DNA (DNeasy extraction kit; Qiagen, Valencia, CA) was subjected to real-time PCR screening using four previously published assays designed to detect B. burgdorferi sensu lato (Lyme group) (36), *Borrelia* within the tick-borne relapsing fever (TBRF) group (36), A. phagocytophilum (37), and Babesia microti (38). Samples were positive if they had a cycle threshold (*C_T_*) value of <40 and logarithmic amplification plots (18).

We present *Borrelia* species data that is differentiated into two broad categories. The first group is B. burgdorferi sensu lato (also recognized as the genus *Borreliella* [39–41]), hereafter referred to as Lyme group *Borrelia*, which includes the disease agent most commonly responsible for Lyme disease in the United States, B. burgdorferi *sensu stricto*, and B. mayonii (5, 42). Additionally, this group includes closely related genospecies, e.g., B. americana, B. andersonii, B. bissettiae, B. californiensis, B. carolinensis, and B. kurtenbachii (30, 41–46). In addition to B. burgdorferi *sensu stricto* and *B. mayonii* (5), human infections have been hypothesized to be the result of additional genospecies, e.g., *B. bissettiae*, *B. americana*, and *B. andersonii* (30, 47, 48), but human disease associations remain unclear or untested. Other Lyme group species have yet to be classified as pathogenic; however, it is essential to remember that identification of pathogenic species often lags behind environmental detection (1).

The second group of *Borrelia* includes the TBRF group, which includes several species commonly found in argasid ticks; however, two species of TBRF *Borrelia* have been found in hard ticks enzootic in the United States: B. miyamotoi (*Ixodes* spp.) and *B. lonestari* (Amblyomma americanum) (49). Since this present study is isolated to *Ixodes* spp., B. miyamotoi is the most likely organism since it has been widely observed in both I. scapularis and I. pacificus in the United States (50); therefore, we refer to these data as B. miyamotoi. Additionally, we present pathogen data on A. phagocytophilum, which causes human granulocytic anaplasmosis, and *Bab. microti*, the agent of babesiosis, which was detected using previously described real-time PCR assays specific to these species only (37, 38). A portion of positive Lyme group *Borrelia*, B. miyamotoi, and A. phagocytophilum samples were sequenced for strain typing and quality control purposes.

Samples were sequenced using previously designed primers that target the 16S-23S intergenic spacer (IGS) (*rrs*-*rrlA*) region for *Borrelia* (51) and the 23S-5S intergenic spacer region of A. phagocytophilum (52) using a nested PCR approach. All thermocycler parameters followed the procedures that were previously published (51, 52). Outer reactions were completed in a 25-μl reaction volume using 2× Phusion MasterMix (ThermoFisher, MA) with a 500 nM primer concentration. Before the inner amplification, the outer product was purified using a 1× magnetic bead cleanup, washed with two 70% ethanol washes, and diluted into 12.5 μl of molecular grade water. Amplified samples were sequenced using capillary Sanger sequencing on an ABI 3730 sequencer with forward and reverse reads (EnGGen, Northern Arizona University).

### Analysis.

Pathogen prevalence in ticks (proportion of ticks positive for pathogen) was calculated for each county, allowing for spatial aggregation of ticks. To further characterize pathogen prevalence across census regions, average county prevalence was computed based on the number of *Ixodes* spp. that were collected from each county (e.g., *n* > 0, *n* > 5, *n* > 10, or *n* > 20). The Northeast included New England (Connecticut, Maine, Massachusetts, New Hampshire, Rhode Island, and Vermont) and Mid-Atlantic divisions (New Jersey, New York, and Pennsylvania). The Midwest included the East North Central (Illinois, Indiana, Michigan, Ohio, and Wisconsin) and West North Central divisions (Iowa, Kansas, Minnesota, Missouri, Nebraska, North Dakota, and South Dakota). The South included South Atlantic (Delaware, Florida, Georgia, Maryland, North Carolina, South Carolina, Virginia, District of Columbia, and West Virginia), East South Central (Alabama, Kentucky, Mississippi, and Tennessee), and West South Central (Arkansas, Louisiana, Oklahoma, and Texas) divisions. Finally, the West included the Pacific (Alaska, California, Hawaii, Oregon, and Washington) and Mountain (Arizona, Colorado, Idaho, Montana, Nevada, New Mexico, Utah, and Wyoming) divisions.

To portray county-level prevalence and estimate the confidence of the prevalence estimates, maps were produced with the prevalence reflected as the color, while the opacity of the fill reflected the level of confidence in the estimate (lighter opacities indicating larger confidence intervals, while darker opacities indicate smaller confidence intervals). Prevalence and confidence intervals were produced through the proportions test (prop.test), which was available through the statistical package “R” (version 4.0.5) (53). Rstudio (version 1.4) was used along with the “tidyverse” (54) and “rgdal” (55) packages to create all figures and conduct the data analysis. County and state shapefiles were utilized from the U.S. Census Bureau. County-level human CDC cases were retrieved from the CDC through the National Notifiable Disease Surveillance System (56). Forward and reverse Sanger sequence reads were trimmed and assembled using the “sangeranalyseR” package (57). Assembled sequences were then compared to sequences available through NCBI BLAST to identify pathogen species (58) and were grouped on the basis of the results.
